# Study of Volatile Organic Compounds of Two Table Grapes (cv. Italia and Bronx Seedless) along Ripening in Vines Established in the Aegean Region (Turkey)

**DOI:** 10.3390/plants11151935

**Published:** 2022-07-26

**Authors:** Ozkan Kaya, Melek Incesu, Fadime Ates, Nurhan Keskin, Nicolás Verdugo-Vásquez, Gastón Gutiérrez-Gamboa

**Affiliations:** 1 Erzincan Horticultural Research Institute, Republic of Turkey Ministry of Agriculture and Forestry, Erzincan 24060, Turkey; 2 Department of Food Engineering, Faculty of Agriculture, Ataturk University, Erzurum 25100, Turkey; melekkaya.2534@gmail.com; 3 Manisa Viticulture Research Institute, Republic of Turkey Ministry of Agriculture and Forestry, Manisa 45125, Turkey; fadimeates2@yahoo.com; 4 Faculty of Agriculture, Department of Horticulture, Van Yüzüncü Yıl University, Van 65090, Turkey; keskin@yyu.edu.tr; 5 Centro de Investigación Intihuasi, Instituto de Investigaciones Agropecuarias INIA, Colina San Joaquín s/n, La Serena 1700000, Chile; nicolas.verdugo@inia.cl; 6 Escuela de Agronomía, Facultad de Ciencias, Ingeniería y Tecnología, Universidad Mayor, Temuco 4780000, Chile

**Keywords:** β-ionone, C6 compounds, grape, neutral variety, terpenes

## Abstract

(1) Background: Italia is a seeded grape variety widely cultivated in the Aegean Region in Turkey, whereas Bronx Seedless is a seedless grape variety, preferred by consumers due to its pink berries and interesting flavor. The goal was to study the volatile compounds of these table grapes throughout berry ripeness. (2) Methods: The volatile compounds were analyzed by GC-MS in six different phenological stages (3) Results: Bronx Seedless grapes presented a higher content of seven terpenes, three aldehydes, one fatty acid, three alcohols, one C6 compound, total aldehydes and total alcohols, and a lower content of eleven terpenes, one fatty acid, four esters, one alcohol, four C6 compounds and its total content than Italia table grapes. The concentration of most of the volatile compounds analyzed increased from “begin of berry touch” to “berries ripe for harvest” stages. Terpenes content in both varieties at harvest was lower than 1.0 mg L^−1^. β-ionone presented the highest odor activity value (OAV) in both varieties. Bronx Seedless grapes presented higher OAV for (Z)-3-hexenal and cedrol, and lower hexanal to (E)-2-hexenal ratio than Italia grapes. (4) Conclusions: Both varieties could be classified as neutral aromatical varieties and it is probable that to achieve a better aromatic quality, Bronx Seedless should be harvested later than Italia.

## 1. Introduction

Volatile organic compounds may play a key role as a quality parameter in table grape production due to their contribution to berry flavor [1]. Several families of volatile compounds, including alcohols, terpenes, esters, C_13_ norisoprenoids, C6 compounds, aldehydes, fatty acids, among others, are responsible for the varietal aroma of fruits [2]. These aromatic compounds may influence consumer preference and acceptance and provide important information about the nutritional value of foods [3]. Grapes are a popular fruit consumed worldwide and berry development has a complex dynamic process that involves a series of biochemical changes [1,4]. Since the content of volatile organic compounds changes during berry development (ripening), harvest time is crucial to provide a fruit of high organoleptic quality [1].

Berry development follows a double sigmoid curve, which presents three main stages: from berry formation to lag phase (Stage I), from lag phase to veraison (Stage II) and post-veraison (Stage III) [5]. The first phase is characterized by a period of rapid cell division and the berry expands in volume and accumulates solutes such as tartaric and malic acids but little amounts of sugar [5]. The second phase is characterized by the slow growth of the berry, the embryo within the seeds matures, the seed coat lignifies, and, finally, the berries start to soften [5]. The post-veraison period is defined as the resumption of growth, and the significant accumulation of anthocyanin and sugars is carried out in the berries [5]. The bound glycoside forms of many volatile compounds in grapes, such as C_13_ norisoprenoids, C6 compounds and terpenes have been identified and quantified during these stages [4].

Grape berries are mostly characterized by their terpene composition, predominantly in varieties that are identified by their Muscat aroma [6]. Terpene composition discriminates among muscat, terpenic and neutral profiles in different grape varieties [7]. Thus, grapes are classified according to their free monoterpene concentrations into neutral, non-Muscat aromatic and Muscat varieties [8]. In Muscat grape cultivars, monoterpenes are the primary compounds [9]. Previous studies have reported differences in volatile organic compounds in berries of different grapevine varieties, including also differences in their composition along berry ripening [10,11]. Some recent studies have investigated the evolution of free volatiles from fruit set to berry ripeness in some neutral and non-Muscat aromatic varieties [10,12,13]. Zhang et al. [13] reported that terpenes content decreased from pre-veraison to veraison, probably due to an inactivation in their biosynthetic pathways, whereas Wu et al. [14] showed that at pre-softening, the volatile compounds content was regulated by expansion dilution, and berry terpene concentration significantly decreased, resulting in a minimum terpene content at softening. Due to the above, the planning of the harvest date must consider these aspects, based on the characteristics desired by consumers.

The Italia (*Vitis vinifera* L.) grape variety is widely grown in Marmara, Aegean, Central Anatolia and Southeastern Anatolia regions in Turkey. Italia is a seeded variety characterized by a mid-late ripeness, a vigorous vegetative behavior and a slight Muscat flavor of berries. Bronx Seedless is an interesting variety and preferred by consumers due to its pink berries that are characterized by their strawberry flavor. To our knowledge, there is little available information about the characterization of berry volatile composition of table grapes along berry ripeness, even more in varieties such as Italia and Bronx Seedless. Thus, the aim of this research was to study the changes in varietal volatile organic compounds in Italia and Bronx Seedless table grapes throughout berry ripeness.

## 2. Results

### 2.1. Content of Terpenes along Ripening in Italia and Bronx Seedless Table Grapes

Table 1 shows the content of some terpenes of Italia and Bronx Seedless table grapes obtained in six phenological stages of vine ripening. The variety factor significantly affected the content of several terpenes, except β-pinene and terpinolene, whereas the phenological stage factor affected the content of most of the terpenes apart from β-pinene. None of the terpenes were affected by the variety and phenological stage interaction factor. Italia grapes showed lower contents of α-pinene, phellandrene, D-limonene and P-cymene, and higher contents of β-myrcene, γ-Terpinene, (E)-rose oxide I and nerol oxide than Bronx Seedless grapes. Generally, grapes harvested at the BBCH-85 and BBCH-89 stages reached higher contents of D-limonene, P-cymene, terpinolene, (E)-rose oxide I and nerol oxide than the grapes harvested in the rest of the phenological stages. Grapes harvested at the BBCH-89 stage showed a higher content of α-pinene than the grapes harvested in the rest of the phenological stages. Contrary to this, grapes obtained at BBCH-77 stages showed a higher content of β-myrcene than the grapes harvested at the BBCH-83, BBCH-85 and BBCH-89 stages.

### 2.2. Content of Other Terpenes along Ripening in Italia and Bronx Seedless Table Grapes

Table 2 shows the content of other terpenes of Italia and Bronx Seedless table grapes obtained in six phenological stages of vine ripening. The variety factor significantly affected the content of several terpenes, except citronellol, geraniol and (E)-nerolidol, whereas the phenological stage factor affected the content of most of the terpenes apart from terpinen-4-ol. Neral was the only terpene affected by the variety and phenological stage interaction. Italia grapes showed higher contents of linalool, terpinene-4-ol, hotrienol, neral, α-terpineol and myrtenol, and lower contents of geranial and nerol than Bronx Seedless. Grapes harvested at the BBCH-89 stage presented higher contents of linalool, hotrienol, neral, α-terpineol and geranial compared to the grapes harvested at the rest of the phenological stages. Generally, grapes harvested at BBCH-89 showed higher contents of citronellol, myrtenol, nerol, geraniol, and (E)-nerolidol than the berries harvested in most of the phenological stages, except BBCH-83 and BBCH-85. Bronx Seedless grapes harvested at the BBCH-77 stage presented a lower content of neral than most of the variety and phenological stage interactions, except for Bronx Seedless, harvested at BBCH-79 (Table S1). Italia grapes harvested at the BBCH-89 stage showed the highest content of neral compared to the rest of the combinations of variety and phenological stage interactions (Table S1).

### 2.3. Content of Some Terpenes, β-ionone, and Fatty Acids along Ripening in Italia and Bronx Seedless Table Grapes

Table 3 shows the content of some other terpenes, β-ionone and fatty acids of Italia and Bronx Seedless table grapes obtained in six phenological stages of vine ripening. The most abundant terpenes found in Italia and Bronx Seedless grapes were geranyl acetone, (E)-nerolidol, cedrol, myrtenol and nerol, whereas the most abundant fatty acid was hexanoic acid (Table 1, Table 2 and Table 3). Variety factor significantly affected the contents of cedrol, geranic acid, geranyl acetone, hexanoic acid and 2-hexenoic acid. The phenological stage factor significantly affected all terpenes, β-ionone and fatty acids shown in Table 3. None of the volatile compounds were affected by the interaction between variety and phenological stage factors. Italia grapes presented lower contents of cedrol and 2-hexenoic acid, and higher contents of geranic acid, geranyl acetone and hexanoic acid compared to Bronx Seedless grapes. Grapes harvested at the BBCH-89 stage presented the highest content of octanoic acid compared to the rest of the phenological stages. These grapes also presented higher contents of cedrol, β-damascenone, geranyl acetone, β-ionone, hexanoic acid and 2-hecenoic acid compared to the grapes harvested at most of the stages except BBCH-85. Grapes harvested at BBCH-77 presented a lower content of geranic acid than the grapes harvested at BBCH-85 and BBCH-89.

### 2.4. Content of C6 Compounds along Ripening in Italia and Bronx Seedless Table Grapes

Table 4 shows the content of C6 compounds of Italia and Bronx Seedless table grapes obtained in the six phenological stages of vine ripening. The most abundant C6 compounds presented in Italia and Bronx Seedless grapes were hexanal and hexanol (Table 4). The variety factor significantly affected the content of several C6 compounds, except (Z)-3-hexenal and (Z)-3-hexenol, whereas the phenological stage affected most of the C6 compounds except (Z)-3-hexenal and (E)-2-hexenol. None of the C6 compounds were affected by the variety and phenological stage interaction. Italia grapes showed higher contents of hexanal, (E)-2-hexenal, hexanol, (E)-3-hexenol and (E)-2-hexenol than Bronx Seedless grapes. Grapes harvested at BBCH-89 presented higher contents of hexanal, (E)-2-hexenal, hexanol, (E)-3-hexenol and (Z)-3-hexenol compared to the grapes harvested in the rest of the phenological stages.

### 2.5. Content of Alcohols along Ripening in Italia and Bronx Seedless Table Grapes

Table 5 shows the content of alcohols of Italia and Bronx Seedless table grapes obtained in the six phenological stages of vine ripening. The most abundant alcohols presented in Italia and Bronx Seedless grapes were 1-octen-3-ol and 2-heptanol (Table 5). Variety factor significantly affected the contents of 1-octen-3-ol, heptanol, nonanol and phenylethyl alcohol, whereas phenological stage factor affected the contents of 2-heptanol, 1-octen-3-ol, octanol, nonanol and benzyl alcohol. None of the alcohols were affected by the interaction between variety and phenological stage factors. Italia grapes showed lower contents of 1-octen-3-ol, heptanol and phenylethyl alcohol, and higher contents of nonanol than Bronx Seedless grapes. Grapes harvested at BBCH-89 stage presented higher contents of 2-heptanol, 1-octen-3-ol, octanol and nonanol than the grapes harvested in the rest of the phenological stages. In addition, the grapes harvested at the BBCH 89 stage presented a higher content of benzyl alcohol than the grapes harvested at the rest of the phenological stages, except BBCH-85.

### 2.6. Content of Esters along Ripening in Italia and Bronx Seedless Table Grapes

Table 6 shows the content of esters of Italia and Bronx Seedless table grapes obtained in the six phenological stages of vine ripening. The variety factor significantly affected the contents of ethyl acetate, ethyl isobutyrate, butyl acetate and ethyl hexanoate, whereas phenological stage factor affected the content of all the esters presented in Table 6. None of the esters were affected by the interaction between variety and phenological stage factors. Italia grapes presented higher contents of ethyl acetate, ethyl isobutyrate, butyl acetate and ethyl hexanoate than the Bronx Seedless grapes. Grapes harvested at the BBCH-89 stage presented the highest content of ethyl propionate compared to the studied stages. In addition, grapes harvested at BBCH-89 showed higher contents of ethyl propionate and ethyl butyrate than the grapes harvested in the rest of the stages, except BBCH-85. BBCH-77 showed lower contents of propyl acetate, ethyl 3-methyl-butanoate, ethyl pentanoate, ethyl hexanoate and hexyl acetate, and a higher content of butyl acetate than the grapes harvested at BBCH-85 and BBCH-89.

### 2.7. Content of Other Esters and Aldehydes along Ripening in Italia and Bronx Seedless Table Grapes

Table 7 shows the content of esters and aldehydes of Italia and Bronx Seedless table grapes obtained in the six phenological stages of vine ripening. The most abundant esters found in Italia and Bronx Seedless grapes were ethy-3-hydroxybutyrate, ethyl octanoate and ethyl heptanoate, whereas the most abundant aldehydes in the studied grapes were benzaldehyde, nonanal and octanal. The variety factor significantly affected the contents of 3-methyl-butanal, pentanal, octanal and nonanal, whereas the phenological factor affected the contents of most of the esters and aldehydes presented in Table 6, except 3-methyl-butanal and benzaldehyde. None of the esters and aldehydes presented in Table 7 were affected by the interaction between variety and phenological stage factors. Italia grapes presented lower contents of 3-methylbutanal, pentanal, octanal and nonanal than Bronx Seedless grapes. Grapes harvested at the BBCH-89 stage presented a higher ethyl octanoate content than the grapes harvested in the rest of the phenological stages. In addition, grapes harvested at the BBCH-77 stage presented lower contents of the esters and aldehydes presented in Table 7 than the grapes harvested at the BBCH-89 stage.

### 2.8. Total Content of C6 Compounds, Alcohols, Esters, Fatty Acids, Aldehydes and Terpenes along Ripening in Italia and Bronx Seedless Table Grapes

**Figure 1:**
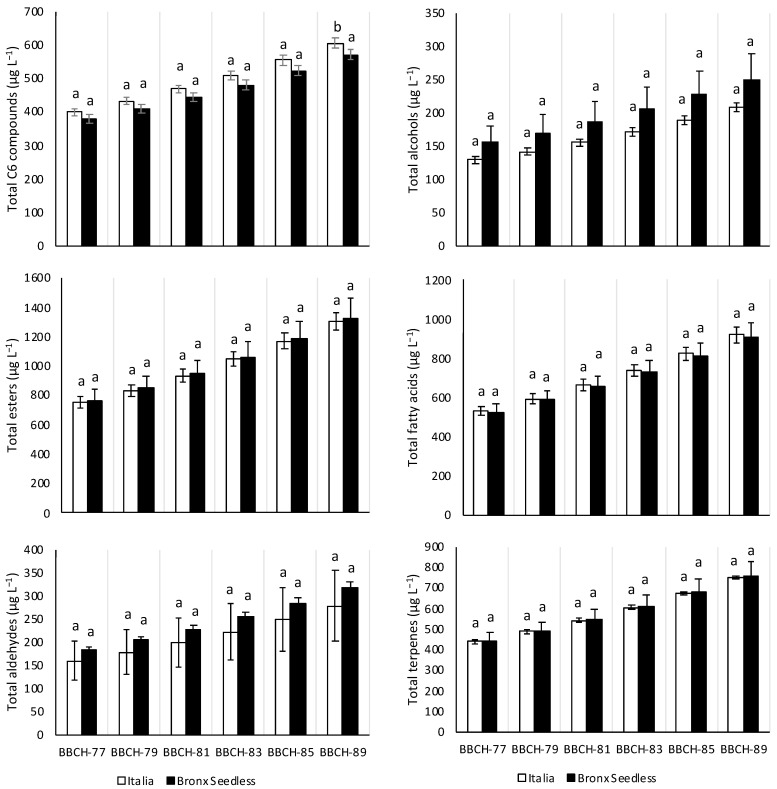
Total content of C6 compounds, alcohols, esters, fatty acids, aldehydes and terpenes in Italia (white) and Bronx Seedless (black) table grapes harvested in six different phenological stages (BBCH-77, BBCH-79, BBCH-81, BBCH-83, BBCH-85 and BBCH-89). Data are expressed as mean of the data with their corresponding standard deviation. For a given variable and phenological stage, different letters over the bars represent significant differences (Duncan test, *p* < 0.05).

Figure 1 shows the total content of C6 compounds, alcohols, esters, fatty acids, aldehydes and terpenes obtained in Italia and Bronx Seedless grapes in the six different phenological stages. The most abundant family of volatile organic compounds were esters, followed by fatty acids, terpenes and C6 compounds. In this way, the most abundant individual volatile organic compounds found in Italia and Bronx Seedless were esters and fatty acids, such as hexanoic acid, ethyl 3-hydroxybutyrate, ethyl octanoate, octanoic acid and 2-hexenoic acid (Table 1, Table 2, Table 3, Table 4, Table 5, Table 6 and Table 7). The total content of C6 compounds, alcohols, esters, fatty acids, aldehydes and terpenes did not differ between the varieties along ripening except at the BBCH-89 stage. In this way, Italia grapes presented higher total C6 compounds content than Bronx Seedless grapes at the BBCH-89 stage.

### 2.9. Odor Activity Values (OAV) of Volatile Compounds Detected and Quantified in Italia and Bronx Seedless Table Grapes at Harvest

Table 8 shows the concentration of volatile compounds at harvest and their corresponding odor activity value (OAV), including their odor threshold and aroma descriptor. Based on the OAVs, β-ionone, which contributes to the balsamic, rose and violet aromatic profile of wines, presented the highest OAV, followed by ethyl isobutyrate (fruity), ethyl 3-methylbutanoate (fruity), (Z)-3-hexenal (grass) and cedrol (cool and canphor). The lowest OAVs were found in propyl acetate (celery), ethyl acetate (pineapple, fruity, solvent, anise and balsamic), benzyl alcohol (roasted, toasted, sweet and fruity), neral (fruity) and nerol oxide (oil and flower) compounds. Italia presented higher OAVs of β-ionone, ethyl isobutyrate and ethyl 3-methylbutanoate, and lower OAVs of (Z)-3-hexenal and cedrol than Bronx Seedless table grapes.

## 3. Discussion

To our knowledge, there is little available information about the study of the volatile composition of table grapes in the scientific literature, neither is there a local contribution from Turkey, whose viticultural production is of the utmost importance as a world supplier. Based on the results exposed, β-ionone presented the highest odor activity value (OAV) in Italia and Bronx Seedless table grapes, which coincided with the findings exposed by Wu et al. [15]. These authors concluded that β-ionone and octanal may be useful indicators for the improvement of breeding in table grapes. Bronx Seedless grapes presented a higher OAV for (Z)-3-hexenal and cedrol than Italia grapes. These volatile compounds present aromatic descriptors related to green aromas, such as grass and camphor (Table 8). Based on this, it is probable that to achieve better aromatic quality, Bronx Seedless should be harvested later than Italia.

Bronx Seedless is characterized by its foxy taste, whereas Italia by its Muscat aroma. Methyl anthranilate is considered the most important contributor to its foxy aroma, but this compound is not necessarily identified by SPME due to its high boiling point and low volatility [12,16]. In this way, methyl anthranilate was not detected in Bronx Seedless and Italia table grapes in the present study. Wu et al. [12] reported that foxy aroma varieties presented lower contents of D-limonene, (E)-β-ocimene, geraniol, α-terpineol, neral, terpinolene, geranic acid, linalool, nerol, citronellol, geranial, (Z)-β-ocimene, nonanal and rose oxide II (cis), and higher contents of ethyl benzoate, methyl salicylate, benzyl alcohol, ethyl 3-hydroxybutyrate, ethyl heptanoate, ethyl pentanoate, ethyl hexanoate, 2-hexenoic acid ethyl ester, ethyl acetate, phenylacetaldehyde, ethyl butyrate, ethyl 3-methylbutanoate, ethyl propionate and ethyl octanoate than Muscat aromatic varieties. The current results show that only α-terpineol, neral, geranic acid and linalool presented these differences between Bronx Seedless (foxy taste) and Italia (Muscat taste) variety. Ruiz-García et al. [17] reported that rose oxide is the key compound to identify and select varieties with a Muscat aroma. Bronx Seedless grapes presented a lower content of rose oxide I (*trans*) than Italia grapes. Terpene content at commercial harvest (BBCH-89) in Italia and Bronx Seedless table grapes was lower than 1.0 mg L^−1^ (749.44 ± 10.56 and 757.23 ± 67.34 μg L^−1^, respectively). In this way, the grapevine varieties could be grouped into Muscat (≥6 mg L^−1^), non-Muscat aromatic (1–4 mg L^−1^) and neutral varieties (<1 mg L^−1^), regarding their monoterpene levels [8]. Despite the above-mentioned, the Italia and Bronx Seedless varieties could be considered as aromatically neutral based on their terpene levels.

The results exposed showed that esters, fatty acids, terpenes and C6 compounds were the most abundant families of volatile compounds in Italia and Bronx Seedless table grapes, in which hexanoic acid, ethyl 3-hydroxybutyrate, ethyl octanoate, 2-hexenoic acid and octanoic acid exhibited the most important concentrations. Regarding OAVs, the most important family of volatile compounds were C_13_-norisoprenoids, followed by esters and C6 compounds. Wu et al. [15] reported that C6 compounds, terpenes and C_13_ norisoprenoids were the main contributors to the aroma in different table grapes obtained from a vineyard located in Shanghai, China. Ubeda et al. [18] reported that C6 compounds were the most abundant family of volatile compounds in new seedless table grapes (Timco^TM^, Magenta^TM^, Krissy^TM^ and Arra15^TM^), followed by aldehydes and ketones. C6 compounds derive from linoleic and linolenic acid and have been described to contribute to the green flavor in table grape berries [12,15]. Hexanal, hexanol and (E)-2-hexenal were the major C6 compounds found in Bronx Seedless and Italia varieties (Table 4 and Table 8). The hexanal to (E)-2-hexenal ratio was correlated to sweetness, an important attribute for table grape production [19]. In this way, (E)-2-hexenal was positively correlated to sourness and crunchiness and negatively to neutral flavor and sweetness [19]. As ripening advanced from BBCH-77 to BBCH-89, this ratio decreased (1.67–1.66, respectively), whereas Italia grapes showed a higher hexanal-to-(E)-2-hexenal ratio than Bronx Seedless grapes (1.69–1.54, respectively).

The concentration of most of the volatile compounds analyzed increased as ripening progressed, except (Z)-3-hexenal, (E)-2-hexenol, heptanol, phenylethyl alcohol, butyl acetate, β-pinene, β-myrcene, rose oxide II (cis) and 4-terpineol (Table 1, Table 2, Table 3, Table 4, Table 5, Table 6 and Table 7). Maoz et al. [19] mentioned that the concentration of some volatile compounds, such as 1-hexanal, 1-hexanol, methyl hexanoate, 1-nonanal, benzaldehyde, rose-oxide, and linalool, often increases during berry development. Contrary to this, the concentration of other volatile compounds, such as geraniol, (E)-2-octenal, and 1-pentanal, often decreases during berry ripening, whereas the levels of other volatile compounds, such as 1-decanal, (E)-2-hexenal, 1-heptanal, geraniol, 2-phenylethanol, and β-ionone, have been suggested to depend on the variety [19]. Yang et al. [10] reported that alcohols, carbonyls, C6 compounds and terpenoids were found in high amounts before veraison, while most of the esters were detected at or after veraison. C6 compounds increased in the early period of berry ripening and then decreased [10].

## 4. Conclusions

The results showed that Bronx Seedless table grapes presented higher contents of α-pinene, phellandrene, D-limonene, P-cymene, geranial, nerol, cedrol, 3-methylbutanal, octanal, nonanal, total aldehydes, 2-hexenoic acid, 1-octen-3-ol, heptanol, phenylethyl alcohol, total alcohols and (E)-3-hexenol, and lower contents of β-myrcene, γ-terpinene, rose oxide I (*trans*), nerol oxide, linalool, 4-terpineol, hotrienol, neral, α-terpineol, myrtenol, geranic acid, hexanoic acid, ethyl acetate, ethyl isobutyrate, butyl acetate, ethyl hexanoate, nonanol, hexanal, (E)-2-hexenal, hexanol, (E)-2-hexenol and total C6 compounds than Italia table grapes. The concentration of most of the volatile compounds analyzed increased from BBCH-77 to BBCH-89, except (Z)-3-hexenal, (E)-2-hexenol, heptanol, phenylethyl alcohol, butyl acetate, β-pinene, β-myrcene, rose oxide II (*cis*) and 4-terpineol. Based on terpene content, Italia and Bronx Seedless varieties could be classified as neutral varieties. β-ionone presented the highest odor activity value (OAV) in Italia and Bronx Seedless table grapes. Bronx Seedless table grapes presented higher OAVs for (Z)-3-hexenal (grass) and cedrol (camphor) than Italia grapes. In addition, Italia grapes showed a higher hexanal-to-(E)-2-hexenal ratio than Bronx Seedless grapes. Therefore, it is probable that to achieve a better aromatic quality, Bronx Seedless should be harvested later than Italia.

## 5. Materials and Methods

### 5.1. Plant Material and Sample Preparation

The research was performed during the 2021 season on twenty-year-old Italia (Bicane × Muscat Hamburg) and Bronx Seedless (New York 8536 × Sultanina) vines grafted onto 5 BB rootstock in the Manisa Viticulture Research Institute, Manisa Province, Aegean Region, Turkey (38°37′57.14″ North Latitude and 27°23′ 57.36″ East Longitude at an altitude of 31.3 m.a.s.l.). Italia is a seeded (formation of seeds) variety and Bronx seedless is a seedless (no formation of seeds) variety. Spur-pruned vines were trained to a high trunk (about 1 m) cordon trellis system with one bunch per shoot and 12–15 shoots per vine at a north–west orientation. Italia and Bronx Seedless vines were planted at a distance of 3.0 m × 2.0 m. Exactly 450 healthy berries from both varieties were randomly harvested in triplicate from both sides of six clusters per vine. Sampling began in the first week before veraison (27 July) and the latest sampling was performed at harvest (28 August), accounting for a total of six sampling phases during berry ripeness. Sampling was performed following different growth stages of the grapevines, according to a BBCH scale published by Lorenz et al. [20]. Grapes were harvested differentially in six different states as follows: BBCH-77 (begin of berry touch), BBCH-79 (berry touch complete), BBCH-81 (berries begin to brighten in color), BBCH-83 (berries brightened), BBCH-85 (softening of berries) and BBCH-89 (berries ripe for harvest), each following the sampling time. Grape samples were harvested and put into bags, then transported immediately in refrigerated conditions (2 to 5 °C) to the laboratory and stored at −80 °C until further analyses.

### 5.2. Chemicals and Reagents

Methanol HPLC grade from Merck (Darmstadt, Germany), NaCl (analytical grade) was obtained from Sigma–Aldrich (Millipore, Bedford, MA, USA) and pure water was obtained from the Milli-Q purification system (Millipore, Bedford, MA, USA). The standards (purity > 95%) used for the quantification and identification of the volatile compounds and the chemical standards were obtained as follows: (E)-2-hexenal, geranic acid, (Z)-3-hexenal, 2-octanol, hexanal, phellandrene, β-damascenone, β-myrcene, D-limonene, benzyl alcohol, citronellol, neral, geranial, α-terpineol, (Z)-rose oxide II, geraniol, (E)-rose oxide I and phenylethyl alcohol from Sigma (St. Louis, MO, USA); hexanoic acid, benzaldehyde, octanoic acid, octanal, nonanal, pentanal, 3-methylbutanal, 1-octen-3-ol, (Z)-3-hexenol, heptanol, ethyl butyrate, ethyl acetate, butyl acetate, ethyl pentanoate, ethyl isobutyrate, methyl salicylate, (E)-2-hexenoic acid, hexyl acetate, terpinolene, P-cymene, linalool, geranyl acetone, 4-terpineol, β-ionone and ethyl hexanoate was obtained from Nu-Chek Prep (Elysian, MN, USA); hexanol, octanol, (E)-2-hexenol from Chem Service Inc. (West Chester, PA, USA). The automatic solid phase microextraction (SPME) fibers of 50/30 μm divinylbenzene/polydimethylsiloxane/carboxen (DVB/PDMS/CAR) were obtained from Supelco (Bellefonte, PA, USA).

### 5.3. Extraction of Volatile Compounds

Volatile organic compounds from the two grape variety samples were extracted by headspace solid phase micro-extraction (HS-SPME) and determined by gas chromatography–mass spectrometry (GC–MS) based on the methodology exposed by Lan et al. [21]. After removing stems and seeds, 120 g of each grape berry sample were mashed and de-seeded and then blended with 1 g polyvinylpolypyrrolidone (PVPP). Subsequently, the obtained samples were crushed by treatment with liquid nitrogen and centrifuged to obtain a clear juice after maceration at 4 °C for 4 h. After this procedure, 5 mL of grape juice were added into a 20 mL flask containing a magnetic stirrer, previously mixed with 10 μL of internal standard (4-methyl-2-pentanol, 1.0018 mg mL^−1^) and 1 g NaCl. Then, the samples were stirred at 40 °C for 30 min on a heating agitation platform. After this, the pre-treated SPME fiber (50/30 μm DVB/Carboxen/PDMS, Supelco, Bellefonte, PA, USA) was inserted into the headspace and extracted for another 30 min with continued agitation and heating. For the GC/MS analysis, the fiber was instantly desorbed into the GC injector.

Free volatile compounds were detected in grapes directly using the above juice. The aroma precursors for the samples were extracted using adsorption on a Cleanert PEP-SPE cartridge (150 mg 6 mL^−1^; Bonna-Agela Technologies, Wilmington, DE, USA) and then eluted by using methanol. The methanolic elutions were concentrated to dryness and then reconstituted in 100 μL of the glycosidase AR2000, and 5 mL of 0.2 mol L^−1^ citrate–phosphate-buffered solution (pH 5.0) (Rapidase, DSM Food Specialties, Delft, The Netherlands).

### 5.4. Analysis of Volatile Compounds by GC-MS 

The oven temperature was programmed as follows: 50 °C for 4 min, increased to 220 °C at 5 °C min^−1^, and then ramped at 20 °C min^−1^ to 220 °C and kept in these conditions for 5 min. The operating conditions in the identification and quantification were determined as follows: ion source, 230 °C, interface, 280 °C and injector, 250 °C. The GC inlet was set in the splitless mode and helium was the carrier gas (1 mL min^−1^). Six independent detections were calculated for each sample from three replicates. Then, retention indices were calculated by analyzing a C6–C24 *n*-alkane series (Supelco) under the same chromatographic conditions. Volatile compounds for samples were identified according to their retention indices and their mass spectra matching in the standard NIST08 library. Quantification procedure for samples was performed according to a previous report published by Wu et al. [22]. Calibration curves obtained from samples were determined with regression coefficients above 98%. The volatile compounds without calibration curves were semi-quantitative determined using the internal standard. Odor activity values (OAV) of each volatile compound were calculated by dividing the concentration of the compound by its odor threshold obtained from the literature [12,15].

### 5.5. Statistical Analysis

The variables were analyzed considering a complete randomized design with factorial arrangement, accounting for two varieties in six phenological stages. The variables were subjected to a multifactorial analysis (MANOVA) that was performed using the Statgraphics Centurion XVI.I (The Plains, VA, USA) statistical package. The significance of the differences was determined by Duncan’s test (*p* ≤ 0.05).

## Figures and Tables

**Table 1:** Terpenes content (μg L^−1^) of Italia and Bronx Seedless table grapes harvested in six different phenological stages (BBCH-77, BBCH-79, BBCH-81, BBCH-83, BBCH-85 and BBCH-89).

	α-Pinene	β-Pinene	β-Phellandrene	β-Myrcene	D-Limonene	γ-Terpinene	o-Cymene	Terpinolene	(E)-Rose Oxide I	Nerol Oxide
**Variety (V)**										
Italia	7.46 a	28.33 a	31.30 a	6.94 b	11.82 a	41.83 b	21.54 a	2.65 a	12.52 b	1.16 b
Bronx Seedless	8.79 b	33.92 a	36.92 b	6.64 a	13.91 b	38.44 a	24.25 b	2.54 a	9.50 a	1.07 a
**Phenological stage (S)**										
BBCH-77	6.13 a	23.48 a	25.09 a	7.14 c	9.42 a	30.28 a	16.97 a	1.96 a	8.31 a	0.84 a
BBCH-79	6.82 a	26.11 a	28.15 ab	7.00 bc	10.58 a	33.67 b	18.99 b	2.18 b	9.24 b	0.94 b
BBCH-81	7.58 ab	29.04 a	31.59 abc	6.86 abc	11.90 b	37.44 c	21.25 c	2.42 c	10.27 c	1.04 c
BBCH-83	8.43 abc	32.29 a	35.44 bc	6.72 ab	13.37 c	41.63 d	23.78 d	2.70 d	11.42 d	1.16 d
BBCH-85	9.37 bc	35.91 a	39.76 cd	6.59 ab	15.03 d	46.29 e	26.61 e	3.00 e	12.70 e	1.29 e
BBCH-89	10.42 c	39.93 a	44.61 d	6.45 a	16.89 e	51.48 f	29.78 f	3.33 f	14.13 e	1.44 f
**Significance ^a^**										
V	0.0375	0.2018	0.0193	0.0109	0.0000	0.0000	0.0000	0.0761	0.0000	0.0017
S	0.0037	0.2614	0.0003	0.0105	0.0000	0.0000	0.0000	0.0000	0.0000	0.0000
V × S	0.9994	1.0000	0.9984	1.0000	0.9083	0.8907	0.9516	0.9997	0.0903	0.9962

Data are expressed as mean of the data. ^a^ Significance (*p*-value) of variety (V), phenological stage (S), and V × S interactions. For a given factor and significance (*p* < 0.05), different letters within a column represent significant differences (Duncan test, *p* < 0.05).

**Table 2:** Content (μg L^−1^) of other terpenes of Italia and Bronx Seedless table grapes harvested in six different phenological stages (BBCH-77, BBCH-79, BBCH-81, BBCH-83, BBCH-85 and BBCH-89).

	Linalool	Terpinen-4-ol	Hotrienol	Neral	α-Terpineol	Geranial	Citronellol	Myrtenol	Nerol	Geraniol	(E)-Nerolidol
**Variety (V)**											
Italia	2.26 b	1.15 b	11.50 b	1.25 b	2.42 b	1.65 a	21.86 a	74.48 b	64.52 a	21.95 a	97.85 a
Bronx Seedless	2.17 a	1.05 a	9.26 a	0.79 a	1.73 a	1.80 b	21.49 a	61.98 a	72.35 b	23.17 a	98.18 a
**Phenological stage (S)**											
BBCH-77	1.59 a	1.16 b	7.83 a	0.77 a	1.56 a	1.24 a	16.35 a	51.47 a	49.09 a	17.02 a	70.30 a
BBCH-79	1.80 b	1.14 ab	8.71 ab	0.86 b	1.74 b	1.40 ab	18.18 ab	57.24 ab	55.57 a	18.93 a	79.59 ab
BBCH-81	2.04 c	1.12 ab	9.68 bc	0.95 c	1.93 c	1.58 bc	20.22 bc	63.65 abc	62.90 ab	21.05 ab	90.09 ab
BBCH-83	2.31 d	1.09 ab	10.77 c	1.06 d	2.15 d	1.79 c	22.48 cd	70.78 bc	71.21 bc	23.41 abc	101.98 bc
BBCH-85	2.61 e	1.07 ab	11.97 d	1.18 e	2.39 e	2.03 d	25.00 de	78.71 cd	80.60 cd	26.03 bc	115.44 cd
BBCH-89	2.96 f	1.06 a	13.31 e	1.31 f	2.66 f	2.30 e	27.80 e	87.52 d	91.24 d	28.94 c	130.68 d
**Significance ^a^**											
V	0.0370	0.0003	0.0000	0.0000	0.0000	0.0190	0.6669	0.0049	0.0472	0.4956	0.9583
S	0.0000	0.1419	0.0000	0.0000	0.0000	0.0000	0.0000	0.0002	0.0000	0.0064	0.0001
V × S	0.9986	1.0000	0.8762	0.0194	0.0681	0.9978	1.0000	0.9971	0.9991	1.0000	1.0000

Data are expressed as mean of the data. ^a^ Significance (*p*-value) of variety (V), phenological stage (S), and V × S interactions. For a given factor and significance (*p* < 0.05), different letters within a column represent significant differences (Duncan test, *p* < 0.05).

**Table 3:** Content of some terpenes, β-ionone, and fatty acids (μg L^−1^) of Italia and Bronx Seedless table grapes harvested in six different phenological stages (BBCH-77, BBCH-79, BBCH-81, BBCH-83, BBCH-85 and BBCH-89).

	Cedrol	Geranic Acid	β-Damascenone	Geranyl Acetone	β-Ionone	Hexanoic Acid	2-Hexenoic Acid	Octanoic Acid
**Variety (V)**								
Italia	70.74 a	29.35 b	26.82 a	108.60 b	27.16 a	418.68 b	135.20 a	154.73 a
Bronx Seedless	83.58 b	17.64 a	26.46 a	91.66 a	26.15 a	381.63 a	166.04 b	154.81 a
**Phenological stage (S)**								
BBCH-77	58.22 a	17.73 a	20.01 a	75.54 a	20.11 a	391.89 a	114.02 a	111.02 a
BBCH-79	64.73 ab	19.71 ab	22.36 ab	84.00 ab	22.36 ab	335.71 ab	126.78 ab	125.67 ab
BBCH-81	71.98 bc	21.92 abc	24.87 bc	93.41 bc	24.87 bc	373.71 bc	140.98 abc	142.26 bc
BBCH-83	80.04 cd	24.38 abc	27.65 cd	103.87 cd	27.65 cd	415.12 cd	156.77 bc	161.03 cd
BBCH-85	89.01 de	27.11 bc	30.75 de	115.50 de	30.75 de	461.61 de	174.33 cd	182.29 d
BBCH-89	98.98 e	30.13 c	34.20 e	128.44 e	34.20 e	513.31 e	193.86 d	206.36 e
**Significance ^a^**								
V	0.0004	0.0000	0.7664	0.0003	0.3282	0.0436	0.0036	0.9894
S	0.0001	0.0347	0.0000	0.0000	0.0000	0.0000	0.0004	0.0000
V × S	0.9892	0.9674	0.8894	0.9882	1.0000	0.9995	0.9964	1.0000

Data are expressed as mean of the data. ^a^ Significance (*p*-value) of variety (V), phenological stage (S), and V × S interactions. For a given factor and significance (*p* < 0.05), different letters within a column represent significant differences (Duncan test, *p* < 0.05).

**Table 4:** C6 compounds content (μg L^−1^) of Italia and Bronx Seedless table grapes harvested in six different phenological stages (BBCH-77, BBCH-79, BBCH-81, BBCH-83, BBCH-85 and BBCH-89).

	Hexanal	(Z)-3-Hexenal	(E)-2-Hexenal	Hexanol	(E)-3-Hexenol	(Z)-3-Hexenol	(E)-2-Hexenol
**Variety (V)**							
Italia	121.06 b	68.55 a	71.62 b	102.20 b	53.63 a	41.26 a	37.09 b
Bronx Seedless	104.54 a	70.33 a	63.81 a	93.89 a	61.42 b	41.41 a	32.90 a
**Phenological stage (S)**							
BBCH-77	82.98 a	72.99 a	49.56 a	73.97 a	42.64 a	31.19 a	36.79 b
BBCH-79	93.11 b	71.53 a	55.71 b	82.25 b	47.71 a	34.68 a	36.05 ab
BBCH-81	104.46 c	70.10 a	62.62 c	91.47 c	53.39 b	38.56 b	35.33 ab
BBCH-83	117.21 d	68.70 a	70.38 d	101.71 d	59.75 c	42.88 c	34.62 ab
BBCH-85	131.51 e	67.33 a	79.11 e	113.10 e	66.85 d	47.68 d	33.93 ab
BBCH-89	147.55 f	65.98 a	88.91 f	125.76 f	74.81 e	53.03 e	33.25 a
**Significance ^a^**							
V	0.0000	0.4277	0.0000	0.0001	0.0000	0.8794	0.0001
S	0.0000	0.4766	0.0000	0.0000	0.0000	0.0000	0.2108
V × S	0.0943	1.0000	0.8862	0.9803	0.9613	1.0000	1.0000

Data are expressed as mean of the data. ^a^ Significance (*p*-value) of variety (V), phenological stage (S), and V × S interactions. For a given factor and significance (*p* < 0.05), different letters within a column represent significant differences (Duncan test, *p* < 0.05).

**Table 5:** Alcohols content (μg L^−1^) of Italia and Bronx Seedless table grapes harvested in six different phenological stages (BBCH-77, BBCH-79, BBCH-81, BBCH-83, BBCH-85 and BBCH-89).

	2-Heptanol	1-Octen-3-ol	Heptanol	2-Ethyl Hexanol	Octanol	Nonanol	Benzyl Alcohol	Phenylethyl Alcohol
**Variety (V)**								
Italia	33.31 a	55.69 a	23.39 a	10.87 a	3.58 a	10.44 b	13.18 a	15.24 a
Bronx Seedless	33.80 a	66.68 b	27.00 b	15.47 a	3.39 a	9.62 a	12.06 a	31.06 b
**Phenological stage (S)**								
BBCH-77	25.31 a	43.89 a	26.49 a	9.93 a	2.63 a	7.57 a	9.06 a	17.47 a
BBCH-79	28.15 b	49.68 ab	25.96 a	11.05 a	2.93 ab	8.42 b	10.25 a	19.42 a
BBCH-81	31.30 c	56.24 b	25.44 a	12.29 a	3.25 b	9.36 c	11.60 ab	21.60 a
BBCH-83	34.81 d	63.67 c	24.93 a	13.66 a	3.62 c	10.40 d	13.13 bc	24.01 a
BBCH-85	38.71 e	72.07 d	24.43 a	15.19 a	4.02 d	11.57 e	14.86 cd	26.71 a
BBCH-89	43.05 f	81.58 e	23.94 a	16.90 a	4.47 e	12.87 f	16.83 d	29.70 a
**Significance ^a^**								
V	0.5170	0.0000	0.0465	0.0686	0.0612	0.0000	0.1418	0.0000
S	0.0000	0.0000	0.9570	0.5738	0.0000	0.0000	0.0000	0.2663
V × S	1.0000	0.9188	1.0000	0.9997	0.9997	0.9163	0.9998	0.9744

Data are expressed as mean of the data. ^a^ Significance (*p*-value) of variety (V), phenological stage (S), and V × S interactions. For a given factor and significance (*p* < 0.05), different letters within a column represent significant differences (Duncan test, *p* < 0.05).

**Table 6:** Esters content (μg L^−1^) of Italia and Bronx Seedless table grapes harvested in six different phenological stages (BBCH-77, BBCH-79, BBCH-81, BBCH-83, BBCH-85 and BBCH-89).

	Ethyl Acetate	Ethyl Propionate	Ethyl Isobutyrate	Propyl Acetate	Ethyl Butyrate	Ethyl 3-Methylbutanoate	Butyl Acetate	Ethyl Pentanoate	Ethyl Hexanoate	Hexyl Acetate
**Variety (V)**										
Italia	25.44 b	23.77 a	26.32 b	26.84 a	35.93 a	22.68 a	13.00 b	23.73 a	10.88 b	24.70 a
Bronx Seedless	16.99 a	22.85 a	20.36 a	31.19 a	34.14 a	21.16 a	10.47 a	22.26 a	8.33 a	22.54 a
**Phenological stage (S)**										
BBCH-77	15.22 a	17.59 a	16.74 a	21.89 a	26.43 a	10.90 a	20.04 c	17.35 a	7.25 a	17.82 a
BBCH-79	17.23 ab	19.56 ab	18.95 a	24.35 ab	29.39 ab	19.29 b	8.06 a	19.29 a	8.06 ab	19.81 ab
BBCH-81	19.50 b	21.75 bc	21.45 ab	27.07 ab	32.69 abc	21.45 bc	8.96 a	21.45 ab	8.96 ab	22.03 abc
BBCH-83	22.07 c	24.18 cd	24.28 bc	30.10 abc	36.35 bc	23.85 bcd	9.97 ab	23.85 abc	9.97 abc	24.50 abc
BBCH-85	24.99 d	26.89 de	27.49 cd	33.47 bc	40.42 cd	26.53 cd	11.08 ab	26.53 bc	11.08 bc	27.24 bc
BBCH-89	28.28 e	29.90 e	31.17 d	37.22 c	44.94 d	29.50 d	12.32 b	29.50 c	12.32 c	30.30 c
**Significance ^a^**										
V	0.0000	0.3900	0.0003	0.1025	0.4021	0.3856	0.0071	0.4225	0.0360	0.3475
S	0.0000	0.0000	0.0000	0.0197	0.0003	0.0000	0.0000	0.0067	0.0106	0.0367
V × S	0.2222	1.0000	0.9740	0.9998	1.0000	1.0000	0.9967	1.0000	0.9965	1.0000

Data are expressed as mean of the data. ^a^ Significance (*p*-value) of variety (V), phenological stage (S), and V × S interactions. For a given factor and significance (*p* < 0.05), different letters within a column represent significant differences (Duncan test, *p* < 0.05).

**Table 7:** Esters and aldehydes content (μg L^−1^) of Italia and Bronx Seedless table grapes harvested in six different phenological stages (BBCH-77, BBCH-79, BBCH-81, BBCH-83, BBCH-85 and BBCH-89).

	Ethyl Heptanoate	Ethyl Octanoate	Ethyl 3-Hydroxybutyrate	2-Methylbutanal	3-Methylbutanal	Pentanal	Octanal	Nonanal	Benzaldehyde	Phenylacetaldehyde
**Variety (V)**										
Italia	119.97 a	236.91 a	375.09 a	25.63 a	23.45 a	17.58 a	30.61 a	33.33 a	37.56 a	26.43 a
Bronx Seedless	128.00 a	239.57 a	403.06 a	30.00 a	30.79 b	25.98 b	35.94 b	42.67 b	33.78 a	24.30 a
**Phenological stage (S)**										
BBCH-77	93.54 a	170.89 a	293.53 a	20.99 a	22.18 a	16.27 a	25.23 a	27.88 a	24.95 a	19.14 a
BBCH-79	104.02 ab	193.44 a	326.41 a	23.34 ab	23.96 a	18.16 ab	28.01 a	31.31 a	28.49 a	21.28 a
BBCH-81	115.67 abc	218.98 b	362.97 ab	25.95 ab	25.88 ab	20.26 ab	31.09 ab	35.16 ab	32.53 ab	23.67 ab
BBCH-83	128.62 abc	247.88 c	403.62 bc	28.86 abc	27.95 ab	22.61 abc	34.51 abc	39.49 abc	37.15 ab	26.31 bc
BBCH-85	143.02 bc	280.60 d	448.83 cd	32.09 bc	30.18 ab	25.23 bc	38.30 bc	44.35 bc	42.43 ab	29.26 cd
BBCH-89	159.05 c	317.64 e	499.10 d	35.68 c	32.59 b	28.16 c	42.52 c	49.8 c	48.45 b	32.54 d
**Significance ^a^**										
V	0.5008	0.6860	0.1586	0.0931	0.0016	0.0002	0.0431	0.0053	0.4163	0.1005
S	0.0343	0.0000	0.0000	0.0227	0.0685	0.0142	0.0050	0.0033	0.0604	0.0000
V × S	1.0000	1.0000	0.9999	0.9998	0.9988	0.9832	0.9995	0.9957	1.0000	0.9988

Data are expressed as mean of the data. ^a^ Significance (*p*-value) of variety (V), phenological stage (S), and V × S interactions. For a given factor and significance (*p* < 0.05), different letters within a column represent significant differences (Duncan test, *p* < 0.05).

**Table 8:** Concentration of volatile compounds at harvest (μg L^−1^) of Italia and Bronx Seedless table grapes and their corresponding odor activity value (OAV), odor threshold (μg L^−1^) and aroma descriptor found in the literature.

Volatile Compounds	Italia	OAV Italia	Bronx Seedless	OAV Bronx Seedless	Odor Threshold ^a^	Aroma Descriptor
***Terpenes***						
α-Pinene	9.57	1.60	11.27	1.88	6.0	Pine, resinous
β-Pinene	36.34	0.26	43.51	0.31	140	Woody, resinous
β-Phellandrene	40.94	1.02	48.29	1.21	40	Terpeny, fruity, minty, herbal
β-Myrcene	6.59	0.18	6.31	0.18	36	Green burning, green
D-Limonene	15.52	1.55	18.26	1.83	10	Fruity, lemon
γ-Terpinene	53.65	0.05	49.31	0.05	1000	Fruity, lemon like
*o*-Cymene	28.01	2.46	31.54	2.77	11.4	Citrus, green
Terpinolene	3.40	0.02	3.26	0.02	200	Piney
(Z)-Rose oxide II	15.26	30.52	16.71	33.42	0.5	Floral, lychee-like, rose
(E)-Rose oxide I	16.06	32.12	12.19	24.38	0.5	Rose
Nerol oxide	1.49	0.00	1.38	0.00	3000	Oil, flower
Linalool	3.01	0.50	2.90	0.48	6.0	Citrus, floral, sweet, grape-like
4-Terpineol	1.10	0.01	1.00	0.01	130	Flowers, nutmeg, moldy
Hotrienol	14.75	0.13	11.87	0.11	110	Fresh, floral, fruity
Neral	1.61	0.00	1.01	0.00	1000	Fruity
α-Terpineol	3.10	0.01	2.22	0.01	330	Lilac, floral, sweet
Geranial	2.40	0.08	2.20	0.07	32	Citrus, citric fruit
Citronellol	28.04	0.70	27.56	0.69	40	Rose
Myrtenol	95.54	13.65	79.50	11.36	7	Flowery, mint
Nerol	86.02	0.03	96.46	0.03	3000	Flower, grass, floral, green
Geraniol	28.16	0.70	29.72	0.74	40	Citric, floral, orange flower, roses, geranium
E-Nerolidol	130.46	0.52	130.9	0.52	250	Rose, apple, green, citrus, waxy, woody
Cedrol	90.74	181.48	107.22	214.43	0.5 *	* Cool, camphor
Geranic acid	37.65	0.94	22.63	0.57	40	Green
***C_13_-norisoprenoids***						
Geranyl acetone	139.30	2.32	117.57	1.96	60	Fresh, floral
β-Ionone	34.84	4977.14	33.55	4792.45	0.007	Balsamic, rose, violet
***Fatty acids***						
Hexanoic acid	537.07	0.18	489.55	0.16	3000	Rancid, cheese, fatty, sweat
2-Hexenoic acid	174.72	0.17	212.99	0.21	1000	Fatty, rancid
Octanoic acid	206.30	0.07	206.41	0.07	3000 *	* Rancid, cheese, fatty, sweat
***C6 compounds***						
Hexanal	158.35	35.19	136.74	30.39	4.5	Green
(Z)-3-Hexenal	65.13	260.52	66.83	267.32	0.25	Grass
(E)-2-Hexenal	94.04	5.53	83.78	4.93	17.0	Grass, herbaceous
Hexanol	131.09	0.26	120.44	0.24	500	Flower, green, cut grass, grass, herbaceous, wood
(E)-3-Hexenol	69.74	0.07	79.88	0.08	1000	Green, bitter, fatty, herbaceous, fresh
(Z)-3-Hexenol	52.92	0.76	53.12	0.76	70	Grass, herbaceous, green, fatty, bitter
(E)-2-Hexenol	35.24	0.35	31.26	0.31	100	Herbaceous, green
***Alcohols***						
2-Heptanol	42.73	0.61	43.36	0.62	70	Fruity, herbaceous
1-Octen-3-ol	74.25	74.25	88.91	88.91	1.0	Mushroom
Heptanol	22.23	0.05	25.65	0.06	425	Oily
2-Ethyl hexanol	13.95	0.05	19.84	0.07	270	Floral
Octanol	4.59	0.04	4.36	0.04	110	Jasmine, lemon
Nonanol	13.39	0.27	12.34	0.25	50	Rose-orange
Benzyl alcohol	17.57	0.00	16.09	0.00	10,000	Roasted, toasted, sweet, fruity
Phenylethyl alcohol	19.55	0.02	39.84	0.04	1100	Floral, rose, honey
***Esters***						
Ethyl acetate	33.92	0.01	22.65	0.00	5000	Pineapple, fruity, solvent, anise, balsamic
Ethyl propionate	30.49	3.05	29.32	2.93	10.0	Banana, apple
Ethyl isobutyrate	35.09	350.9	27.14	271.43	0.1	Fruity
Propyl acetate	34.43	0.01	40.01	0.01	4700	Celery
Ethyl butyrate	46.10	46.10	43.79	43.79	1.0	Fruity
Ethyl 3-methylbutanoate	30.44	304.40	28.55	285.50	0.1	Fruity
Butyl acetate	13.96	0.21	10.68	0.160	66	Fruity
Ethyl pentanoate	30.44	20.29	28.55	19.03	1.5	Grass
Ethyl hexanoate	13.96	13.96	10.68	10.68	1.0	Fruity, green apple, banana, wine-like, brandy
Hexyl acetate	31.68	0.05	28.91	0.04	670	Apple, floral, fruity, banana, pear, brandy
(Z)-3-Hexenyl acetate	57.72	0.08	55.63	0.07	750 *	* Fruity, green leaves
Ethyl heptanoate	153.89	76.95	164.19	82.09	2.0	Winelike, brandy, fruity
Ethyl octanoate	315.87	1.63	319.41	1.65	194	Sweet, floral, fruity, banana, pear, brandy
Ethyl 3-hydroxybutyrate	481.16	0.02	517.03	0.03	20,000	Grape, fruity, caramel, toasted
***Aldehydes***						
2-Methylbutanal	32.88	25.29	38.48	29.60	1.3 *	* Green, malty
3-Methylbutanal	28.18	140.90	37.00	185.02	0.2 *	* Fresh grass, cocoa
Pentanal	22.73	1.89	33.59	2.80	12.0	Fat, green
Octanal	39.11	55.87	45.92	65.60	0.7	Honey, green, fatty, fruity, citrus, lemon, fat, soap
Nonanal	43.68	43.68	55.92	55.92	1.0	Fat, citrus, green, fruity
(E)-2-Octenal	26.19	8.73	28.67	9.56	3.0 *	* Green, nut, fat
Benzaldehyde	51.02	0.15	45.89	0.13	350	Sweet, fruity, roasted, almond, fragrant, burnt sugar
Phenylacetaldehyde	33.90	8.48	31.17	7.79	4.0	Flowery, rose

^a^ Expressed in μg L^−1^. * Information obtained from the report published by Wu et al. [15]. The rest of the presented information about odor threshold and aroma descriptor of each volatile compound was obtained from the data published by Wu et al. [12].

## Data Availability

Not applicable.

## Supplementary Materials

The following supporting information can be downloaded at: https://www.mdpi.com/article/10.3390/plants11151935/s1, Table S1. Concentration of neral (μg L^−1^) during the interaction between variety (V) and phenological stage (S) factors.
