# Supplementary material for: Study of Volatile Organic Compounds of Two Table Grapes (cv. Italia and Bronx Seedless) along Ripening in Vines Established in the Aegean Region (Turkey)
Source: Plants (Basel). 2022 Jul 26;11(15):1935. doi: 10.3390/plants11151935 (PMC9329889; doi:10.3390/plants11151935)
Supplement: Supplementary file 1 [file plants-11-01935-s001.zip › plants-1825847-supplementary.pdf]

Supplementary material

Table S1. Concentration of neral ($\mu g\ L^{-1}$) of the interaction between variety (V) and phenological stage (S) factors.

Interaction	Neral
Bronx Seedless BBCH-77	0.60 ± 0.02 a
Bronx Seedless BBCH-79	0.66 ± 0.02 ab
Bronx Seedless BBCH-81	0.74 ± 0.02 bc
Bronx Seedless BBCH-83	0.82 ± 0.03 cd
Bronx Seedless BBCH-85	0.91 ± 0.03 de
Italia BBCH-77	0.95 ± 0.06 ef
Bronx Seedless BBCH-89	1.01 ± 0.03 ef
Italia BBCH-79	1.05 ± 0.07 f
Italia BBCH-81	1.17 ± 0.08 g
Italia BBCH-83	1.30 ± 0.09 h
Italia BBCH-85	1.45 ± 0.10 i
Italia BBCH-89	1.61 ± 0.11 j

Data are expressed as mean of the data. [a]Significance (*p*-value) of variety (V), phenological stage (S), and V x S interactions. Different letters within a column represent significant differences (Duncan test, $p < 0.05$).
